# Neuronal GPCR OCTR-1 regulates innate immunity by controlling protein synthesis in *Caenorhabditis elegans*

**DOI:** 10.1038/srep36832

**Published:** 2016-11-11

**Authors:** Yiyong Liu, Durai Sellegounder, Jingru Sun

**Affiliations:** 1 Department of Biomedical Sciences, Elson S. Floyd College of Medicine, Washington State University, Spokane, Washington, USA

## Abstract

Upon pathogen infection, microbial killing pathways and cellular stress pathways are rapidly activated by the host innate immune system. These pathways must be tightly regulated because insufficient or excessive immune responses have deleterious consequences. Increasing evidence indicates that the nervous system regulates the immune system to confer coordinated protection to the host. However, the precise mechanisms of neural-immune communication remain unclear. Previously we have demonstrated that OCTR-1, a neuronal G protein-coupled receptor, functions in the sensory neurons ASH and ASI to suppress innate immune responses in non-neural tissues against *Pseudomonas aeruginosa* in *Caenorhabditis elegans*. In the current study, by using a mass spectrometry-based quantitative proteomics approach, we discovered that OCTR-1 regulates innate immunity by suppressing translation and the unfolded protein response (UPR) pathways at the protein level. Functional assays revealed that OCTR-1 inhibits specific protein synthesis factors such as ribosomal protein RPS-1 and translation initiation factor EIF-3.J to reduce infection-triggered protein synthesis and UPR. Translational inhibition by chemicals abolishes the OCTR-1-controlled innate immune responses, indicating that activation of the OCTR-1 pathway is dependent on translation upregulation such as that induced by pathogen infection. Because OCTR-1 downregulates protein translation activities, the OCTR-1 pathway could function to suppress excessive responses to infection or to restore protein homeostasis after infection.

The nematode *Caenorhabditis elegans* is a powerful model system for infection studies in the context of a whole animal. More than 40 microbes have been shown to be pathogenic to *C. elegans*, including bacteria, fungi and viruses, some of which are also human pathogens^1^. Unlike vertebrates or many other invertebrate species, *C. elegans* does not have an adaptive immune system; it relies on innate immunity and avoidance behavior to defend itself against microbial attacks. Upon pathogen infection, the nematode can mount protective responses by triggering evolutionarily conserved signaling cascades. These cascades include the mitogen-activated protein kinase (MAPK) pathways, the DAF-2/insulin-like receptor pathway, the DBL-1 pathway (homologous to the mammalian TGF-β cascade), the unfolded protein response (UPR), and programmed cell death^2^,^3^,^4^,^5^. Activation of these signaling pathways induces expression of defensive genes. The most common differentially expressed protein families in *C. elegans* during pathogenesis include C-type lectins, lysozymes, lipases and antimicrobial peptides^1^,^2^,^6^. These molecules are regarded as markers of immune responses because they are positively regulated in various organisms exposed to a broad range of pathogens. They are believed to be the immune effectors that act directly to fight off infection, although only a few markers have been demonstrated to have such activities^7^,^8^,^9^.

Upon pathogen infection, cellular stress pathways and microbial killing pathways are rapidly activated by the host innate immune system. These pathways must be tightly regulated as insufficient immune responses exacerbate infection, whereas excessive immune responses lead to prolonged inflammation, tissue damage and death. For example, in *C. elegans*, loss of the XBP-1-dependent UPR in the presence of pathogens leads to disruption of morphology of the endoplasmic reticulum (ER) and larval lethality^10^, while under physiological conditions results in constitutive ER stress^11^. Hyperstimulation of the p38 MAPK pathway is also toxic to *C. elegans*^12^. In humans, dysregulated innate immune responses have been linked to a myriad of human health concerns such as sepsis, autoimmune disease and chronic inflammatory disease. Increasing evidence indicates that the nervous system regulates the immune system to confer coordinated protection to the host^13^,^14^,^15^. Recent studies on neuronal G protein-coupled receptors (GPCRs) highlight the roles of specific neurons in the regulation of immune responses^16^,^17^,^18^,^19^. *C. elegans* deficient in NPR-1, a homologue of the neuropeptide Y receptor in mammals, shows decreased innate immune responses to infections by *Pseudomonas aeruginosa*, *Salmonella enterica* or *Enterococcus faecalis* but enhanced immune defense against *Bacillus thuringiensis*^16^,^19^. This suggests that the NPR-1-expressing neurons regulate innate immunity to certain pathogens. We have demonstrated that loss of expression of OCTR-1, a putative catecholamine GPCR, in the nematode’s sensory ASH and ASI neurons increases innate immune responses to *P. aeruginosa*, indicating that OCTR-1 functions in ASHs and ASIs to suppress innate immunity^17^,^18^. This regulation is partially achieved by down-regulating gene expression of the p38 MAPK pathway and the UPR pathways in pharyngeal and intestinal tissues that are the primary line of defense against microbial pathogens. It is currently unknown how the immuno-modulatory signals are relayed from ASH/ASI neurons to the non-neuronal tissues. The above studies investigated transcriptional regulatory mechanisms underlying neural regulation of innate immunity; however, it remains unclear how such regulation operates at the protein level.

Although one might expect a direct correspondence between mRNA transcripts and protein expression^20^, recent studies have shown that the mRNA-protein correlation can be low. Transcriptomic and proteomic studies under similar conditions often reported that only a small overlap exists between differentially regulated genes and proteins^21^,^22^,^23^,^24^,^25^,^26^. There are several reasons for this discordance. First, technological biases lead to a much lower proteome coverage than transcriptome coverage^27^,^28^. The most widely used technologies for transcriptomic profiling are DNA microarray and RNA sequencing. Mass spectrometry techniques are used for proteomic profiling. Technical resolution at the proteome level is much more constrained, resulting in identification of only those proteins with high difference in abundance. Second, physical properties of transcripts influence translation. For example, transcripts with weak Shine-Dalgarno (SD) sequences are translated less efficiently^29^,^30^. Third, codon bias affects the mRNA-protein correlation^31^,^32^. Fourth, ribosome-density, the number of ribosome in a transcriptional unit, has a major influence on efficiency of translation^33^,^34^,^35^. Finally, the correlation between half-lives of mRNAs and proteins is low^36^. For example, post-translational modifications significantly influence the stability of proteins. While RNA can serve as direct biological effectors, proteins are the effectors of most biological functions. Genome-wide analysis at the protein level is, therefore, a more direct reflection of gene expression.

Since the first use of the *P. aeruginosa*-*C. elegans* model for pathogenesis research in 1999^37^,^38^, a number of transcriptomic studies have been carried out to investigate the nematode’s innate immunity^39^,^40^,^41^,^42^. In the current work, we examine how *C. elegans* responds to *P. aeruginosa* infection and how the nervous system regulates those responses at the protein level.

## Results

### *P. aeruginosa* infection induces proteomic changes in *C. elegans*

**Figure 1:**
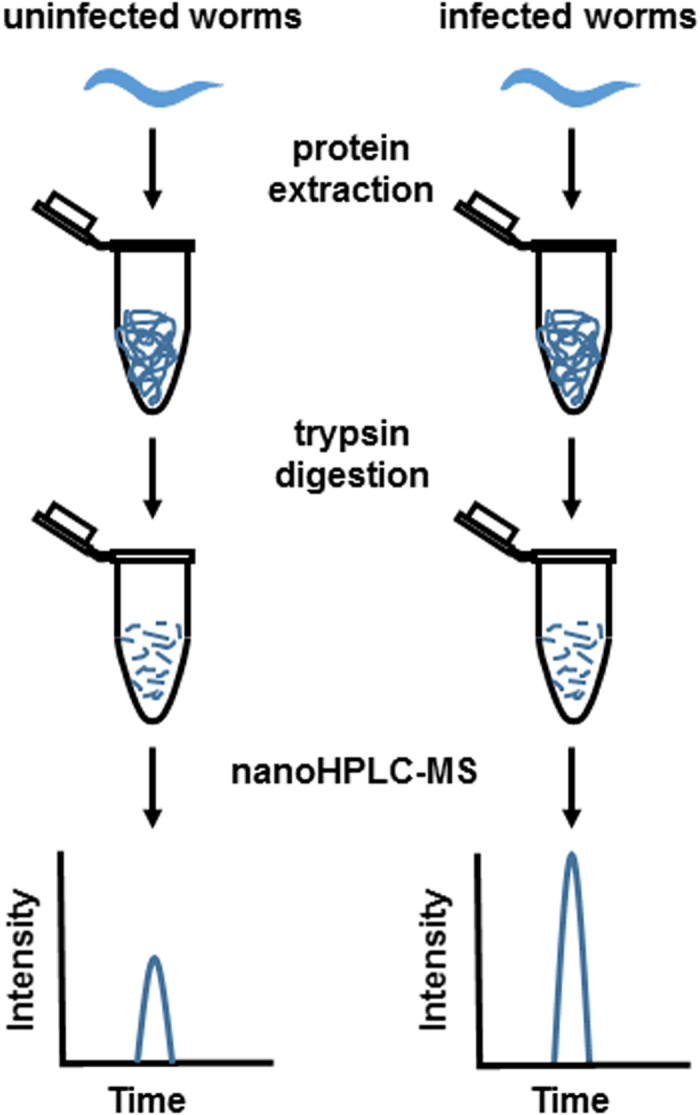
Scheme of label-free quantitative proteomics. Synchronized animals at L4 stage were either fed *E. Coli* OP50 (uninfected controls) or *P. aeruginosa* PA14 (infected samples) for 4 hr, collected, and lysed. Total proteins were extracted and digested with trypsin. Peptides from control or infected samples were subject to nano-HPLC tandem MS analysis. Quantification is based on the comparison of peak intensity of same peptides in different samples.

To investigate how *C. elegans* responds to *P. aeruginosa* infection at the protein level, we compared the proteomes of infected wild-type N2 worms to those of uninfected controls using a label-free quantitative proteomics approach. A schematic diagram of this approach is depicted in Fig. 1. Five biological replicates of each experimental group were individually analyzed with high resolution nano-HPLC tandem mass spectrometry. Protein identification and quantification were done using Thermo Scientific Proteome Discoverer software. In total, 4,413 proteins were identified and quantified, among which 1,312 proteins were identified with high confidence (1% false discovery rate, FDR). The levels of these 1,312 proteins were statistically compared between experimental groups using TIGR Multiexperiment Viewer (MeV). The proteomics data are available via ProteomeXchange with identifier PXD004173.

Compared with the proteins identified in the uninfected wild-type worms, 53 proteins were significantly upregulated at least 1.5-fold in the *P. aeruginosa*-infected wild-type worms (Table 1). Twenty-three of these proteins were only detected in the infected samples, indicating that their abundance was below the detection limit in the uninfected worms. We performed gene ontology (GO) enrichment analyses to identify significantly enriched biological processes using the web-based program Gorilla (http://cbl-gorilla.cs.technion.ac.il/)^43^. Analysis of the 53 upregulated proteins against a background of 1,312 proteins (the total number of proteins identified with high confidence) revealed 15 enriched biological processes, 14 of which involve the nematode’s responses to infection or other external stimuli (Table 2). For example, the most significantly enriched GO term is “innate immune response” (FDR = 8.7E-09, enriched 9.28-fold); and the highest enriched GO term is “defense response to Gram-negative bacterium” (FDR = 7.08E-05, enriched 12.85-fold) (Table 2). As *P. aeruginosa* is a Gram-negative bacterium and known to trigger innate immune responses in *C. elegans*^39^,^40^,^41^,^44^, these results validate the effectiveness of our proteomics approach for detecting differentially expressed proteins under infection conditions. We also found that 16 proteins were downregulated in *P. aeruginosa*-infected wild-type worms by more than 1.5-fold (Table S1). Gorilla analysis of these 16 proteins yielded no enriched GO terms.

The proteins upregulated by *P. aeruginosa* infection belong to a number of functional groups, as shown in Table 1. These include markers of immunity, such as CUB-like proteins, C-type lectins, lysozymes, proteins containing ShK toxin domain, and glutathione s-transferases (GSTs). Immune markers are a set of genes/proteins that are positively regulated in various organisms in response to a wide range of pathogens; they are believed to be the immune effectors that enhance the host’s ability to fight off pathogens^1^,^2^,^6^,^45^. Many immune markers were discovered by transcription studies. Our finding that marker genes are also induced at the protein level suggests that these genes are biologically functional during immune response. When compared with three whole-genome transcription studies in the literature^39^,^40^,^41^, which used microarray to detect differentially expressed genes in *C. elegans* upon *P. aeruginosa* infection, nine out of the 12 marker proteins that were upregulated in our proteomics study are also induced at the transcript level (Table 1). Table 1 also shows the upregulation of a group of 13 proteins that have proteolysis, hydrolysis or oxidoreductase activities, which might reflect increased metabolism in the nematode and the need for extra energy whilst fighting off an infection. About half of these proteins overlap with the induced transcripts revealed in the microarray studies^39^,^40^,^41^. Despite the fact that 70% of the 53 upregulated proteins have no match in the transcriptomic data (Table 1), the two types of studies showed good agreement on the enrichment of innate immune proteins and enzymes involved in macromolecule metabolism.

### OCTR-1 regulates innate immune responses at the protein level

Previously we have shown that neuronally expressed OCTR-1 suppresses *P. aeruginosa*-triggered innate immune responses in *C. elegans* by down-regulating gene expression in the UPR pathways and the p38 MAPK pathway^17^,^18^. These studies were performed at the transcript level. To investigate how OCTR-1 regulates innate immunity at the level of protein expression, we examined the proteomic changes of *octr-1*(*ok371*) worms relative to wild-type worms exposed to *P. aeruginosa*. As shown in Table S2, infection upregulated 113 proteins in the mutant worms. Twenty-three of these are immunity marker proteins, which almost doubles the number of the immune proteins induced in the wild-type worms (Table 3 versus Table 1). The result is consistent with an inhibitory role of OCTR-1 in innate immunity^17^,^18^. Strikingly, all 12 immune proteins induced in wild-type worms are upregulated in *octr-1*(*ok371*) worms, illustrating the high-degree reproducibility of our proteomics method. Besides the immune proteins, we also observed significant induction of proteins in the UPR pathway in *octr-1*(*ok371*) worms (Table 3), demonstrating that OCTR-1 inhibits the UPR at the protein level. This correlates well with our previous genome microarray study that showed OCTR-1 suppresses the UPR at the transcript level^17^,^18^. Knockdown of a number of UPR genes individually (*abu-1, -7, -8, -12, -13, xbp-1*, or *Y41C4A.11*) by RNA interference (RNAi) partially or fully rescued the mutant phenotype of *octr-1*(*ok371*) worms, i.e. decreased the immunity of the mutants to the wild-type level^17^,^18^. Taken together, these results suggest that elevated UPR activity contributes to the enhanced immunity of *octr-1* mutant worms.

Interestingly, we observed significant induction of a number of proteins with functions in transcription, sulfur amino acid biosynthesis and translation in the mutant worms (Table 3). In contrast, abundance of these proteins in the wild-type worms did not change upon infection (Table 1). We attribute the induction of these proteins in the mutants to the lack of OCTR-1. Therefore, in addition to the UPR, OCTR-1 also potentially regulates transcription, sulfur amino acid biosynthesis and translation in response to *P. aeruginosa* infection. Surprisingly, the abundance of the transcripts of these proteins remained unchanged in *octr-1*(*ok371*) relative to wild-type worms in our previous whole-genome microarray study conducted under the same infection conditions^17^, suggesting that induction of these proteins occurs at the post-transcriptional level. Because enhanced transcription, sulfur amino acid biosynthesis and translation likely promote protein synthesis, it is reasonable to speculate that higher protein synthesis activity is responsible for the upregulation of many proteins in *octr-1*(*ok371*) worms, which in turn triggers strong UPR in the mutants.

### OCTR-1 modulates protein synthesis in response to *P. aeruginosa* infection

**Figure 2:**
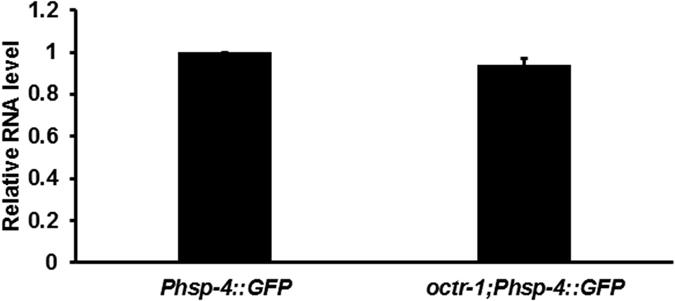
qRT-PCR analysis of *gfp* gene expression in *Phsp-4::GFP*(*zcls4*) and *octr-1*(*ok371*)*;Phsp-4::GFP*(*zcls4*) animals exposed to *P. aeruginosa*. Relative fold-changes for *gfp* transcripts were normalized to *pan-actin* (*act-1, -3, -4*). *Phsp-4::GFP*(*zcls4*) versus *octr-1*(*ok371*)*;Phsp-4::GFP*(*zcls4*), *p* = 0.07. Bars represent mean ± SEM. *n* = 3 independent experiments.

Dunbar *et al*. showed that translation of green fluorescent protein (GFP) was blocked in the intestine of *C. elegans* during *P. aeruginosa* infection through the use of transgene *hsp-16.2::GFP* as a reporter for translation activity^46^. To investigate if OCTR-1 plays a direct role in modulating protein synthesis, we used a similar transgenic GFP reporter *Phsp-4::GFP*(*zcls4*). The *C. elegans hsp-4* gene encodes a homologue of mammalian BiP/GRP-78; and intestinal expression of the transgene *Phsp-4::GFP*(*zcIs4*) has been used as an indicator of the XBP-1-dependent UPR^18^,^47^, which reflects protein accumulation and demand on protein folding in the endoplasmic reticulum. Previously we have demonstrated that upon *P. aeruginosa* infection, *octr-1*(*ok371*)*;Phsp-4::GFP*(*zcls4*) animals exhibited significantly higher levels of GFP expression than *Phsp-4::GFP*(*zcls4*) animals (Fig. 2 in Sun *et al*.^18^). It is unclear whether the elevated GFP expression was due to upregulation of transcription or increased translation or both. To find out the cause of this phenomenon, we performed qRT-PCR to compare the levels of GFP mRNA in *Phsp-4::GFP*(*zcls4*) and *octr-1*(*ok371*)*;Phsp-4::GFP*(*zcls4*) animals exposed to *P. aeruginosa*. As shown in Fig. 2, the levels of GFP mRNA were not significantly different between the two strains, indicating that the elevated GFP expression in the *octr-1* mutants was due to upregulation of translation, not transcription. Hence, OCTR-1 modulates protein synthesis in response to *P. aeruginosa* infection.

### Protein synthesis factors are involved in the OCTR-1-dependent immunity

**Figure 3:**
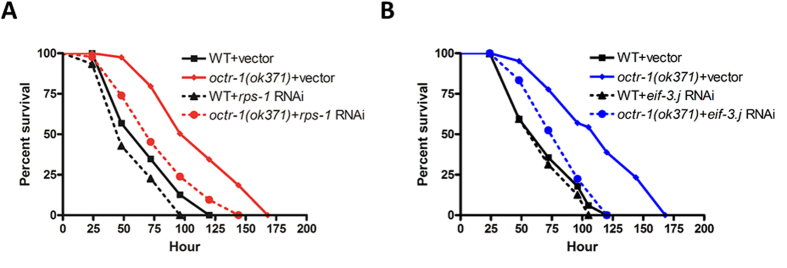
Protein synthesis factors are involved in the OCTR-1-depedent immunity. (**A**) Wild-type and *octr-1*(*ok371*) animals grown on double-stranded RNA (dsRNA) for vector control or dsRNA for *rps-1* were exposed to *P. aeruginosa* PA14 and scored for survival over time. WT+ vector versus WT+*rps-1* RNAi: *p* = 0.0386; *octr-1*(*ok371*)+vector versus *octr-1*(*ok371*)+*rps-1* RNAi: *p* < 0.0001. Shown is a representative assay of three independent experiments. *n* = 45 young adult animals per strain. Significant knockdown of *rps-1* expression by RNAi was confirmed by qRT-PCR (*p* < 0.001, Figure S2A). (**B**) Wild-type and *octr-1*(*ok371*) animals grown on dsRNA for vector control or dsRNA for *eif-3.j* were exposed to *P. aeruginosa* PA14 and scored for survival over time. WT+vector versus WT+*eif-3.j* RNAi: *p* = 0.4873; *octr-1*(*ok371*)+vector versus *octr-1*(*ok371*)+ *eif-3.j* RNAi: *p* = 0.0001. Shown is a representative assay of three independent experiments. *n* = 45 young adult animals per strain. *p* values < 0.05 are considered significant. Significant knockdown of *eif-3.j* expression by RNAi was confirmed by qRT-PCR (*p* < 0.001, Figure S2B).

Eight ribosomal proteins (RPs) were upregulated by *P. aeruginosa* in *octr-1*(*ok371*) animals relative to wild-type animals, including RPS-1, RPS-11, MRPL-12, RPL-13, RPL-18, RPS-10, RPS-28, and K07C5.4 (Table 3). Recent studies revealed the multifunctional roles of RPs ranging from protein synthesis to apoptosis^48^,^49^,^50^. Identification of these upregulated RPs raises an important question: do RPs play any roles in innate immunity? To answer this question, we knocked down the expression of the above RPs individually by RNAi in both wild-type and *octr-1*(*ok371*) animals, then measured the nematode’s survival against *P. aeruginosa* infection, as well as their lifespan on a standard food source *E. coli* OP50. This approach allows us to determine gene contribution to immunity independently of possible functions important for development or lifespan. The RNAi experiment demonstrated that silencing the RPs in many cases led to delay in development or even to maternal sterility (Table S3). The results are in agreement with the previous studies in zebrafish that showed knockdown of many individual RPs causes cell death or defective development^51^,^52^. As components of ribosome, the expression of RPs is tightly regulated to provide the appropriate ratio between RPs and rRNAs as well as among RPs. Thus, perturbation of ribosome biogenesis results in ribosomal stress, which can trigger p53-dependent cell cycle arrest and apoptosis^53^,^54^,^55^. Nonetheless, we observed that RNAi of *rps-1* did not alter the development (Table S3) or lifespan of *C. elegans* (Figure S1), but strongly enhanced susceptibility to *P. aeruginosa* in *octr-1* mutant animals and also exerted a subtle effect on the susceptibility of wild-type animals (Fig. 3A). The result indicates that this RP plays a role in innate immunity of *C. elegans*, which adds value to the extraribosomal functions of RPs.

Besides the above RPs, two more proteins (RUVB-1 and EIF-3.J) involved in protein synthesis were upregulated in *octr-1*(*ok371*) animals compared to wild-type animals upon *P. aeruginosa* infection (Table 3 versus Table 1). RUVB-1 is an AAA+ ATPase orthologous to the RUVBL1 family of ATPases and functions as a component of the Target of Rapamycin (TOR) signaling pathway that enables robust protein synthesis^56^. EIF-3.J is an orthologue of the j-subunit of human translation initiation factor EIF3 that participates in nearly all steps of translation initiation^57^. While RNAi of *ruvb-1* caused developmental delay (Table S3), knockdown of *eif-3.j* increased pathogen susceptibility in *octr-1* mutants, whereas the same knockdown had no effects in wild-type animals (Fig. 3B). This result indicates that EIF-3.J is important for the OCTR-1-controlled immunity.

### Protein synthesis factors RPS-1 and EIF-3.J contribute to the elevated UPR in infected *octr-1*(*ok371*) animals

**Figure 4:**
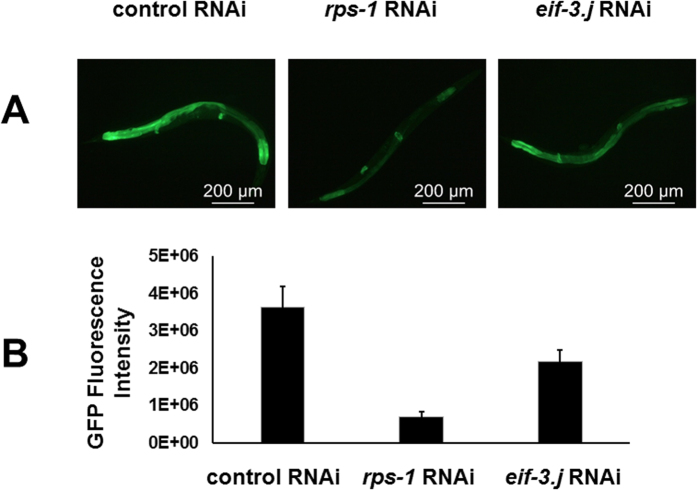
Knockdown of *rps-1* or *eif-3.j* by RNAi reduces the XBP-1-dependent UPR in *octr-1* mutant animals. (**A**) Images of *octr-1*(*ok371*)*;Phsp-4::GFP*(*zcls4*) animals. Animals were grown on double-stranded RNA for vector control or dsRNA for *rps-1* or *eif-3.j*, and young adult animals were exposed to *P. aeruginosa* PA14. Animals that best represent the fluorescence level of the population were shown. (**B**) GFP quantification of *octr-1*(*ok371*)*;Phsp-4::GFP*(*zcls4*) animals exposed to *P. aeruginosa* PA14. Binary mean intensity of the region of interest (ROI) that corresponds to an entire animal was measured by Image J software. *n*  = 10–20 animals, error bars represent SEM. A two-sample *t* test was performed to compare the fluorescence intensity between populations. *rps-1* RNAi versus control RNAi: *p* = 6.1 × 10^−10^; *eif-3.j* RNAi versus control RNAi: *p* = 4.8 × 10^−7^. *p* values < 0.05 are considered significant. Significant knockdown of *rps-1* or *eif-3.j* expression by RNAi was confirmed by qRT-PCR (*p* < 0.001, Figure S3).

Because both RPS-1 and EIF-3.J have important roles in protein translation, lack of either protein is expected to reduce protein synthesis. This would also lower the elevated UPR in *octr-1*(*ok371*) animals exposed to *P. aeruginosa*. To test this prediction, we took advantage of the established correlation between intestinal expression of *Phsp-4::GFP*(*zcls4*) and XBP-1-dependent UPR^18^,^47^, and used *Phsp-4::GFP*(*zcls4*) as a reporter to examine if knockdown of *rps-1* or *eif-3.j* by RNAi could lower the elevated UPR in *P. aeruginosa*-infected *octr-1*(*ok371*)*;Phsp-4::GFP*(*zcls4*) animals^18^. As shown in Fig. 4, RNAi of *rps-1* or *eif-3.j* in infected *octr-1*(*ok371*)*;Phsp-4::GFP*(*zcls4*) animals significantly reduced *Phsp-4::GFP* expression, as compared to the control RNAi with an empty vector. The above result demonstrated that both RPS-1 and EIF-3.J contribute to the elevated UPR in *P. aeruginosa*-infected *octr-1*(*ok371*) animals, which in turn enhances their immunity. Because expression of the same transgene *Phsp-4::GFP*(*zcls4*) also reflects OCTR-1-dependent protein synthesis (Section 3 in Results), the contribution of RPS-1 and EIF-3.J in UPR can be attributed to their protein synthesis activity.

### Translational inhibition by chemicals abolishes the OCTR-1-controlled innate immune responses

**Figure 5:**
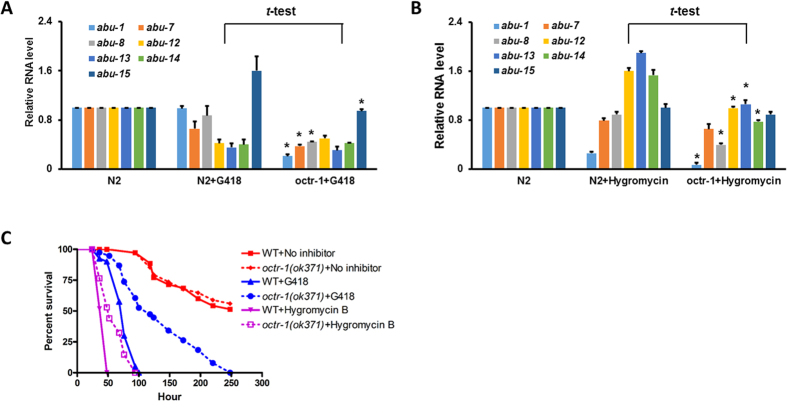
Translational inhibition abolishes the OCTR-1-controlled innate immune responses. (**A**) qRT-PCR of *abu-1*, *abu-7*, *abu-8*, *abu-12*, *abu-13*, *abu-14*, and *abu-15* expression in wild-type animals with or without exposure to G418 and in *octr-1*(*ok371*) animals exposed to G418. *n* = 3; bar graphs correspond to mean ± SEM. *t*-tests were performed between N2 + G418 and *octr-1* + G418. *indicates significant difference. (**B**) qRT-PCR of *abu-1*, *abu-7*, *abu-8*, *abu-12*, *abu-13*, *abu-14*, and *abu-15* expression in wild-type animals with or without exposure to Hygrocymin B and in *octr-1*(*ok371*) animals exposed to Hygromycin B. *n* = 3; bar graphs correspond to mean ± SEM. *t*-tests were performed between N2 + G418 and *octr-1* + G418. *indicates significant difference. (**C**) Wild-type and *octr-1*(*ok371*) animals were exposed to NGM/*E. coli* OP50 (control) or NGM/*E. coli* OP50 containing G418 or Hygromycin B, and scored for survival over time. WT + No inhibitor versus *octr-1*(*ok371*)+No inhibitor: *p* = 0.7521; WT + G418 versus *octr-1*(*ok371*)+G418: *p* < 0.0001; WT + Hygromycin B versus *octr-1*(*ok371*)+Hygromycin B: *p* < 0.0001. Shown is a representative assay of two independent experiments. *n* = 45 young adult animals per strain. *p* values < 0.05 are considered significant.

Translational inhibition is a very common pathogenic attack strategy; consequently, response to such inhibition is a conserved form of host defense^46^,^58^,^59^,^60^,^61^. However, how this response is regulated in the host remains largely unknown. Because OCTR-1 downregulates protein synthesis activities, the OCTR-1 pathway could function to suppress excessive responses to translational inhibition or to restore protein homeostasis after infection. To test this possibility, we treated wild-type and *octr-1*(*ok371*) animals with translation inhibitors G418 and Hygromycin B. These chemicals have been used to mimic *P. aeruginosa*-mediated host translational suppression in *C. elegans*^46^,^58^. We evaluated the effects of such inhibition by assessing: 1) expression levels of the OCTR-1-dependent immune genes; and 2) animal survival against the inhibition. Previously, we have shown that OCTR-1 suppresses innate immunity by downregulating the expression of non-canonical UPR *abu* (activated in blocked unfolded protein response) genes^17^. Here we examined the expression of seven *abu* genes by qRT-PCR, including *abu-1*, *abu-7*, *abu-8*, *abu-12*, *abu-13*, *abu-14* and *abu-15*. As shown in Fig. 5A,B, G418 or Hygromycin B itself had various effects on the expression of *abu* genes in wild-type animals. Specifically, G418 downregulates *abu-7*, *abu-12*, *abu-13* and *abu-14* and upregulates *abu-15*, while Hygromycin B downregulates *abu-1* and upregulates *abu-12*, *abu-13* and *abu-14*. It is unclear why these translational inhibitors differentially regulate the *abu* genes. Nonetheless, a comparison of gene expression levels between G418-treated wild-type and *octr-1*(*ok371*) animals showed that *abu-1*, *abu-7*, *abu-8*, and *abu-15* were significantly downregulated in the *octr-1* mutants (Fig. 5A). Similarly, a comparison of gene expression levels between Hygromycin B-treated wild-type and *octr-1*(*ok371*) animals showed that *abu-1*, *abu-8*, *abu-12*, *abu-13*, and *abu-14* were significantly downregulated in the *octr-1* mutants (Fig. 5B). Interestingly, none of the *abu* genes increased expression in the inhibitor-treated *octr-1*(*ok371*) animals compared to wild-type animals with or without treatments (Fig. 5A,B). These results indicate that activation of the OCTR-1-controlled innate immune responses (i.e. upregulation of *abu* genes in *octr-1* mutants relative to wild-type animals) is dependent on active translation. Despite lack of the OCTR-1-controlled immune responses, *octr-1*(*ok371*) animals are more resistant than wild-type animals to G418 or Hygromycin B treatment, although the chemical treatments shortened the survival time of both strains (Fig. 5C). It is likely that the higher protein translation activities in the mutants offset some of the inhibitory effects of the chemicals, thus allow their longer survival than the wild-type strains. These results suggest that protein translation is important for the OCTR-1-controlled innate immunity.

## Discussion

We have examined the proteomic changes in *C. elegans* upon *P. aeruginosa* infection using a label free quantitative proteomics approach. Fifty-three proteins were significantly upregulated in the infected wild-type worms relative to the uninfected controls. The data are in good agreement with the previously published transcriptomic studies^39^,^40^,^41^ on the enrichment of innate immune proteins and enzymes involved in macromolecule metabolism (Table 1). However, about 70% of the 53 proteins have no match in the transcriptomic data, including a set of nine proteins with known functions in development and a number of proteins with unknown functions (Table 1). Whether these proteins play any roles in immunity or pathogenesis warrant further investigation.

Previously we have demonstrated that OCTR-1 functions in ASH and ASI neurons to suppress transcription of non-canonical UPR genes of the *pqn/abu* family^17^,^18^. In the current proteomics study, we have identified ten PQN proteins, including PQN-22, PQN-24, PQN-27, PQN-32, PQN-41, PQN-51, PQN-52, PQN59, PQN-74 and PQN87. Surprisingly, none of these proteins have known functions in the non-canonical UPR pathway^17^,^62^. Many *abu* and *pqn* genes that were found significantly upregulated in our previous genome microarray study^17^ have not been identified at the protein level in the current proteomics study. This discrepancy is most likely caused by the different technologies used in the two studies. The *C. elegans* GeneChip (Affymetrix, Santa Clara, CA) was used in the genome microarray study, which examines the expression of 22,500 transcripts, while label-free quantitative nano-HPLC tandem mass spectrometry was used in the proteomics study, which only identified 4,413 proteins with 1,312 proteins identified with high confidence. Technical resolution at the proteome level is much more constrained than that at the transcriptome level, leading to a much lower proteome coverage than transcriptome coverage and identification of only those proteins with high difference in abundance^63^.

Consistent with our genome microarray study^17^, here we show that OCTR-1 inhibits translation and the UPR at the protein level. Our RNAi experiments and functional assays support the following conclusions: 1) OCTR-1 inhibits expression of specific protein synthesis factors, such as ribosomal protein RPS-1 and translation initiation factor EIF-3.J, which reduces infection-triggered protein synthesis and UPR; 2) upregulation of UPR proteins and elevated UPR in *octr-1*(*ok371*) animals contribute to their enhanced immunity; 3) RPS-1 and EIF-3.J contribute to the elevated UPR in infected *octr-1*(*ok371*) animals; and 4) activation of the OCTR-controlled immune response is dependent on active translation. The contributions of the upregulated protein synthesis activities to the enhanced immunity of infected *octr-1*(*ok371*) animals are two-fold: 1) higher protein synthesis activities lead to production of more immune proteins, which enhance the nematode’s ability to fight off invading pathogens; and 2) increased protein production results in elevated UPR, which in turn improves the animal immunity. As translational inhibition is a very common pathogenic attack strategy and OCTR-1 has homology in various species including humans, the OCTR-1-dependent pathway may be a conserved signaling pathway that the nervous system uses to control protein homeostasis during host immune defense.

## Materials and Methods

### *C. elegans* and bacterial strains

The following *C. elegans* strains were cultured under standard conditions and fed *E. coli* OP50^64^. Wild-type worms were *C. elegans* Bristol N2. *Octr-1*(*ok371*) and *Phsp-4::gfp* (*zcIs4*) strains were obtained from the *Caenorhabditis elegans* Genetics Center (University of Minnesota, Minneapolis, MN). The mutant *octr-1*(*ok371*)*;Phsp-4::GFP*(*zcIs4*) was constructed using standard genetic techniques^18^. *E. coli* strain OP50 and *P. aeruginosa* strain PA14^44^ were grown in Luria-Bertani (LB) broth at 37 °C.

### *C. elegans* survival assay

*C. elegans* wild-type worms and mutants were maintained as hermaphrodites at 20 °C and fed with *E. coli* OP50 on modified nematode growth medium (NGM) agar plates (0.35% instead of 0.25% peptone) as described^64^. The bacterial lawn used for *C. elegans* killing assays were prepared by placing a 25 μl drop of an overnight culture of the bacterial strains on modified NGM agar plates (3.5 cm diameter Petri plates). Plates were incubated at 37 °C for 16 hr. Plates were cooled down at room temperature for at least 1 hr before seeding with synchronized worms. The survival assays were performed at 25 °C and live worms were transferred daily to fresh plates. Worms were scored at the times indicated and were considered dead when they failed to respond to touch.

### *C. elegans* infection and collection

Synchronized wild-type and *octr-1*(*ok371*) worms grown to L4 larval stage were infected with *P. aeruginosa* at 25 °C for 4 hr, as we described previously^17^. Infected worms and uninfected controls (on *E. coli* OP50) were collected and washed 5 times with M9 buffer in the presence of protease inhibitors (ProteaseArrest, G-Biosciences, St. Louis, MO) to remove surface bound bacteria. In the last wash, after centrifugation and removal of the supernatant, each worm pellet was immediately frozen in liquid nitrogen. Five biological replicates of infected and uninfected worms were collected. The frozen worm samples were submitted to the Tissue Imaging and Proteomics Laboratory at Washington State University (Pullman, WA) for mass spectrometry-based quantitative proteomics analyses.

### Protein sample preparation

The worm pellet was ground into fine powder using a single 2.8 mm i.d. steel ball with a TissueLyser II (Qiagen, Valencia, CA) at a frequency of 30 Hz for 30 sec, followed by the addition of 100 μl extraction solvent of PBS buffer, pH 7.5, with protease inhibitor and vortexing. Supernatants were collected after centrifugation at 16,000 × g (10 min, 4 °C). Protein was then quantified with a Qubit Protein Assay Kit (Life Technologies, Carlsbad, CA) in compliance with the manufacturer’s protocol. Disulfide bonds were reduced using 100 mM dithiothreitol (DTT) at a ratio of 1:10 DTT/sample volume and incubated at 50 °C for 45 min. Cysteine bonds were then alkylated with 200 mM iodoacetamide at the same volume ratio for 20 min at room temperature. Finally, protein was digested with trypsin (G-Biosciences, St. Louis, MO) at a 1:50 ratio of trypsin/protein, and incubated at 37 °C for 12 hr.

### High resolution nano-HPLC tandem mass spectrometry analysis

The peptide samples were subjected to Thermo Scientific Orbitrap Fusion Tribrid with an Easy-nLC 1000 ultra-high pressure LC on a Thermo Scientific PepMap 100 C18 column (2 μm, 50 μm × 15 cm). The peptides were separated over 115 min gradient eluted at 400 nL/min with 0.1% formic acid (FA) in water (solvent A) and 0.1% FA in acetonitrile (solvent B) (5–30% B in 85 min, followed by 30–50% B over 10 min and 50–97% B over 10 min). The run was completed by holding a 97% B for 10 min. MS1 data was acquired on an Orbitrap Fusion mass spectrometry using a full scan method according to the following parameters: scan range 400–1500 m/z, Orbitrap resolution 120,000; AGC target 400,000; and maximum injection time of 50 ms. MS2 data were collected using the following parameters: rapid scan rate, HCD collision energy 35%, 1.6 m/z isolation window, AGC 2,000 and maximum injection time of 50 ms. MS2 precursors were selected for a 3 s cycle. The precursors with an assigned monoisotopic m/z and a charge state of 2–7 were interrogated. The precursors were filtered using a 60 s dynamic exclusion window. MS/MS spectra were searched using Thermo Scientific Proteome Discoverer software version 2.0 with SEQUEST^®^ against uniprot Caenorhabditis elegans database (TaxID = 6239). Precursor and fragment mass tolerances were set to 10 ppm and 0.8 Da respectively and allowing up to two missed cleavages. The static modification used was carbamidomethylation (C). The high confidence level filter with false discovery rate (FDR) of 1% was applied to the peptides. Protein relative quantitation was achieved by extracting peptide areas with the Proteome Discoverer 2.0 (Thermo Scientific, San Jose, CA) and 3 unique peptides per protein were used for the protein quantitation analysis. The mass spectrometry proteomics data have been deposited to the ProteomeXchange Consortium via the PRIDE^65^ partner repository with the dataset identifier PXD004173.

### RNA interference

RNA interference was conducted by feeding *C. elegans* with *E. coli* strain HT115(DE3) expressing double-stranded RNA (dsRNA) that is homologous to the target gene of interest^66^,^67^. Briefly, *E. coli* with the appropriate vectors was grown in LB broth containing ampicillin (100 μg/ml) at 37 °C overnight, and plated onto NGM plates containing 100 μg/ml ampicillin and 3 mM isopropyl β-D-thiogalactoside (IPTG). RNAi-expressing bacteria were allowed to grow overnight at 37 °C. L2 or L3 larval worms were placed on RNAi or vector control plates for 2 days at 20 °C until nematodes became gravid. Gravid adults were then transferred to fresh RNAi-expressing bacterial lawns and allowed to lay eggs for 1 hr at 25 °C to synchronize a second-generation RNAi population. The gravid adults were removed and eggs were allowed to develop at 20 °C to reach young adult stage for subsequent assays. Clone identity was confirmed by sequencing. *unc-22* RNAi was included as a positive control in all experiments to account for RNAi efficiency.

### Fluorescence imaging

Gravid adult *Phsp-4::GFP*(*zcIs4*) or *octr-1*(*ok371*)*;Phsp-4::GFP*(*zcIs4*) were transferred to NGM plates seeded with *E. coli* OP50 for 1 hr at 25 °C to lay eggs. Gravid adults were removed from NGM plates and the eggs were allowed to hatch at 20 °C. Young adults were then exposed to *P. aeruginosa* PA14 or *E. coli* OP50 at 20 °C for 24 hr. Worms were observed under Leica MZ10F microscope and fluorescence images were taken using LAS V4.5 software. Binary mean intensity was measured by ImageJ software.

### Translation inhibitor treatment

Three thousand one hundred twenty five μl of 50 mg/ml G418 (Roche, New York, NY) or 750 μl of 50 mg/ml Hygromycin B (Mediatech, Inc. Manassas, VA) was added to 250 ml NGM medium, respectively, to make corresponding G418 or Hygromycin B plates using 6 cm Petri dishes. 100 ml overnight *E. coli* OP50 cell culture was centrifuged at 4000 rpm for 10 min. Concentrated *E. coli* OP50 was seeded on the above translational inhibitor plates. Synchronized wild-type and *octr-1*(*ok371*) worms at young adult stage were transferred to NGM plates containing *E. coli* OP50 with or without translation inhibitor for survival assays and scored over time. The survival assays were performed at 25 °C and live worms were transferred daily to fresh plates. Worms were scored at the times indicated and were considered dead when they failed to respond to touch.

### RNA isolation

Gravid adult wild-type and *octr-1*(*ok371*) worms were transferred to NGM plates seeded with *E. coli* OP50 for 1 hr at 25 °C to lay eggs. Gravid adults were removed from NGM plates and the eggs were allowed to hatch at 20 °C for 3 days. Young adult worms were then exposed to NGM plates containing *E. coli* OP50 with or without translation inhibitor at 25 °C for 4 hr. After 4 hr, worms were collected and washed with M9 buffer, and RNA was extracted using QIAzol lysis reagent and purified with RNeasy Plus Universal Kit (Qiagen, Valencia, CA).

### Quantitative real-time PCR (qRT-PCR)

Total RNA was obtained as described above and subjected to reverse transcription as suggested by High Capacity cDNA Reverse Transcription Kit (Applied Biosystems, Foster City, CA). Quantitative PCR was conducted on StepOnePlus Real-Time PCR system using Power SYBR Green PCR Master Mix in a 96-well plate format (Applied Biosystems). Fifty nanograms of cDNA were used for real-time PCR. Twenty-five microliter reactions were set-up and performed as outlined by the manufacturer (Applied Biosystems). Relative fold-changes for transcripts were calculated using the comparative *C*_T_ (2^−ΔΔ*C*^_T_) method and normalized to pan-actin (*act-1, -3, -4*). Cycle thresholds of amplification were determined by StepOne Software v2.3 (Applied Biosystems). All samples were run in triplicate. Primer sequences are available on request.

### Statistical Analysis

For the proteomics study, protein levels between experimental groups were statistically compared using TIGR Multiexperiment Viewer v4.9 (MeV, http://www.tm4.org/mev.html). The relative quantities of the proteins were compiled as tab-delimited text files and input into MeV v4.9. *t*-tests were performed between experimental groups with 5 replicates in each group. The program generates 2 text files with significantly different proteins and non-significantly different proteins in separate files along with *P*-values of each comparison. *P*-values < 0.05 are considered significant. For the *C. elegans* survival assays, animal survival was plotted as a non-linear regression curve using the PRISM (version 6, GraphPad Software, Inc. La Jolla, CA) computer program. Survival curves were considered different than the appropriate control indicated in the main text when *P*-values were < 0.05. Prism uses the product limit or Kaplan-Meier method to calculate survival fractions and the logrank test (equivalent to the Mantel-Heanszel test) to compare survival curves. A two-sample *t* test for independent samples was used to analyze qRT-PCR results; *P*-values < 0.05 are considered significant. All the experiments were repeated at least 3 times, unless otherwise indicated.

### Availability of data and material

The mass spectrometry proteomics data of “N2 versus octr-1 shotgun proteomics nano MS/MS” have been deposited to the ProteomeXchange Consortium via the PRIDE partner repository (https://www.ebi.ac.uk/pride/archive/) with the dataset identifier PXD004173.

## Additional Information

**How to cite this article**: Liu, Y. *et al*. Neuronal GPCR OCTR-1 regulates innate immunity by controlling protein synthesis in *Caenorhabditis elegans*. *Sci. Rep*. **6**, 36832; doi: 10.1038/srep36832 (2016).

**Publisher’s note**: Springer Nature remains neutral with regard to jurisdictional claims in published maps and institutional affiliations.

## Supplementary Material

Supplementary Information

## Figures and Tables

**Table 1:** *P. aeruginosa*-induced proteins in wild-type N2 animals.

Functional Group	Protein	Gene ID	Fold Change#	p-Value	Overlap with published microarray data*		
					Troemel *et al*.	Shapira *et al*.	Evan *et al*.
CUB-like domain	DOD-24	*c32h11.12*	only in the infected		x	x	
	F55G11.2	*f55g11.2*	10.1	7.58E-03	x	x	x
	F55G11.4	*f55g11.4*	only in the infected				
	C32H11.4	*c32h11.4*	only in the infected		x	x	x
	DOD-17	*k10d11.1*	only in the infected		x	x	
	C17H12.8	*c17h12.8*	only in the infected		x		x
C-type lectin	CLEC-63	*f35c5.6*	3.4	1.11E-04			
	CLEC-66	*f35c5.9*	only in the infected		x	x	
Lysozyme	LYS-2	*y22f5a.5*	only in the infected			x	
	LMP-1	*c03b1.12*	only in the infected				
ShK toxin domain	C14C6.5	*c14c6.5*	only in the infected			x	x
GST	GST-38	*f35e8.8*	only in the infected		x	x	
Proteolysis/hydrolysis	ASP-14	*k1*^*0c*^*2.3*	3.4	5.97E-04	x	x	
	M60.2	*m60.2*	only in the infected		x		
	BRE-1	*c53b4.7*	2.3	1.18E-03			
	HEX-1	*t14f9.3*	only in the infected				
	CAT-4	*f32g8.6*	only in the infected				
	B0222.5	*b0222.5*	only in the infected				
oxidoreductases	F20D6.11	*f20d6.11*	only in the infected		x		
	VNA	*r04b5.5*	1.8	5.72E-03			
	C55A6.4	*c55a6.4*	only in the infected				
	GLRX-5	*y49e10.2*	3.3	4.56E-03			
	F53C11.3	*f53c11.3*	5.1	2.63E-03	x	x	
	DAF-22	*y57a10c.6*	1.9	7.01E-03	x		
	MAOC-1	*e04f6.3*	2.0	2.93E-03		x	
Heat shock protein	Y55F3BR.6	*y55f3br.6*	2.0	2.25E-03			
	DNJ-19	*t05c3.5*	3.1	2.28E-03			
Development	NASP-2	*c50b6.2*	2.5	6.75E-03			
	Y71F9AL.9	*y71f9al.9*	2.3	5.39E-03			
	CPG-2	*b0280.5*	2.1	6.37E-03			
	MLC-4	*c56g7.1*	2.2	2.14E-03			
	VPS-32.1	*c56c10.3*	2.7	4.38E-03			
	T23D8.3	*t23d8.3*	only in the infected				
	RMD-1	*t05g5.7*	only in the infected				
	SEC-24.2	*zc518.2*	2.1	6.44E-03			
	MRPL-34	*c25a1.13*	only in the infected				
Others or unknown	DSC-4	*k02d7.4*	1.8	9.23E-03			
	CEY-4	*y39a1c.3*	1.7	4.01E-04			
	ZC247.1	*zc247.1*	1.7	4.37E-03			
	PQN-59	*r119.4*	1.7	6.29E-03			
	PUD-2.1	*f15e11.1*	2.6	4.85E-05			
	Y44A6D.2	*y44a6d.2*	1.8	1.59E-03			
	Y69A2AR.18	*y69a2ar.18*	1.7	8.28E-03			
	VDAC-1	*r05g6.7*	only in the infected				
	CLEC-209	*f56a4.2*	only in the infected				
	C15C7.5	*c15c7.5*	4.3	5.37E-04			
	W05H9.1	*w05h9.1*	2.1	3.93E-03			
	GLB-1	*zk637.13*	3.0	5.27E-03		x	
	ZK418.9	*zk418.9*	1.8	4.27E-03			
	LPD-8	*r10h10.1*	2.1	1.34E-03			
	SNA-1	*w02f12.6*	only in the infected				
	IRG-3	*f53e10.4*	only in the infected		x	x	
	LBP-4	*zk742.5*	2.1	8.12E-03			

^#^Only in the infected: the protein was only detected in the infected animals, not in the uninfected animals.

^*^Troemel *et al*. 2006 PLoS Genet 2(11):183; Shapira *et al*. 2006 Proc Natl Acad Sci USA 103(38):14086–14091; Evans *et al*. 2008 PLoS Pahtog 4(10):e1000175.

**Table 2:** GO term (biological process) enrichment analysis of *P. aeruginosa*-induced proteins in wild-type N2 animals.

GO term	Description	P-value^#^	FDR q-value^*^	Enrichment (N, B, n, b)^§^
GO:0002376	immune system process	1.25E-11	2.63E-08	9.28 (1098, 36, 46, 14)
GO:0006955	immune response	1.25E-11	1.32E-08	9.28 (1098, 36, 46, 14)
GO:0045087	innate immune response	1.25E-11	8.77E-09	9.28 (1098, 36, 46, 14)
GO:0006952	defense response	7.98E-11	4.19E-08	7.46 (1098, 48, 46, 15)
GO:0006950	response to stress	7.15E-08	3.00E-05	3.91 (1098, 110, 46, 18)
GO:0050829	defense response to Gram-negative bacterium	2.03E-07	7.08E-05	12.85 (1098, 13, 46, 7)
GO:0050896	response to stimulus	2.27E-07	6.79E-05	3.64 (1098, 118, 46, 18)
GO:0043207	response to external biotic stimulus	1.23E-06	3.22E-04	10.44 (1098, 16, 46, 7)
GO:0098542	defense response to other organism	1.23E-06	2.86E-04	10.44 (1098, 16, 46, 7)
GO:0042742	defense response to bacterium	1.23E-06	2.57E-04	10.44 (1098, 16, 46, 7)
GO:0009617	response to bacterium	1.23E-06	2.34E-04	10.44 (1098, 16, 46, 7)
GO:0009607	response to biotic stimulus	1.23E-06	2.15E-04	10.44 (1098, 16, 46, 7)
GO:0051707	response to other organism	1.23E-06	1.98E-04	10.44 (1098, 16, 46, 7)
GO:0009605	response to external stimulus	3.87E-05	5.80E-03	6.68 (1098, 25, 46, 7)
GO:0051704	multi-organism process	4.65E-04	6.50E-02	3.64 (1098, 59, 46, 9)

^#^P-value is the enrichment p-value computed according to the mHG model (Eden *et al*. 2007 PLos Comp Bio 3(3):e39).

*FDR q-value is the correction of the above p-value for multiple testing using the Benjamini and Hochberg method (Benjamini and Hochberg 1995 J R Statist Soc B 57(1):289–300).

^§^Enrichment (N, B, n, b) is defined as follows:

N - is the total number of proteins.

B - is the total number of proteins associated with a specific GO term.

n - is the number of proteins in the target set.

b - is the number of proteins in the intersection.

Enrichment = (b/n)/(B/N).

**Table 3:** *P. aeruginosa*-induced proteins in *octr-1*(*ok371*) animals.

Biological Functions	Protein	Gene ID	Description	Fold Change#	p-Value
Innate immune response	DOD-24	*c32h11.12*	CUB-like domain	infected only	
	F55G11.2	*f55g11.2*	CUB-like domain	infected only	
	F55G11.4	*f55g11.4*	CUB-like domain	infected only	
	C32H11.4	*c32h11.4*	CUB-like domain	infected only	
	DOD-17	*k10d11.1*	CUB-like domain	infected only	
	CLEC-67	*f56d6.2*	C-type lectin	infected only	
	CLEC-66	*f35c5.9*	C-type lectin	infected only	
	CLEC-63	*f35c5.6*	C-type lectin	3.5	6.05E-03
	SKPO-1	*f49e12.1*	ShK toxin domain	infected only	
	C14C6.5	*c14c6.5*	ShK toxin domain	infected only	
	LYS-2	*y22f5a.5*	lysozyme	infected only	
	GST-38	*f35e8.8*	glutathione S-transferase	infected only	
	GST-5	*r03d7.6*	glutathione S-transferase	9.5	6.98E-03
	GST-4	*k08f4.7*	glutathione S-transferase	2.8	9.81E-03
	GST-7	*f11g11.2*	glutathione S-transferase	3.9	9.31E-03
	GCS-1	*f37b12.2*	glutathione biosynthesis	2.7	8.72E-03
	ASP-14	*k10c2.3*	aspartyl protease	7.2	5.77E-03
	M60.2	*m60.2*	ortholog of human endonuclease, polyU-specific	infected only	
	C17H12.8	*c17h12.8*	involved in innate immune response	infected only	
	F35E12.6	*f35e12.6*	involved in innate immune response	infected only	
	IRG-3	*f53e10.4*	infection response protein	infected only	
	SKR-3	*f44g3.6*	skp1 related (ubiquitin ligase complex component)	2.3	6.32E-03
	DJR-1.1	*b0432.2*	glutathione-independent glyoxalase DJR-1.1	1.8	2.25E-03
Sulfur amino acid biosynthesis	METR-1	*r03d7.1*	probable methionine synthase	infected only	
	C01G10.9	*c01g10.9*	methylthioribose-1-phosphate isomerase	infected only	
	F58H1.3	*f58h1.3*	enolase-phosphatase E1	2.5	8.81E-05
	SAMS-4	*c06e7.3*	probable S-adenosylmethionine synthase 4	1.8	9.82E-03
	CTH-2	*zk1127.10*	putative cystathionine gamma-lyase 2	4.1	6.44E-03
	CYSL-2	*k10h10.2*	bifunctional L-3-cyanoalanine synthase/cysteine synthase	2.3	1.71E-04
	HMG-11	*t05a7.4*	predicted to have DNA binding activity	infected only	
Translation/protein synthesis	RPL-18	*y45f10d.12*	60S ribosomal protein L18	2.1	5.23E-03
	RPS-10	*d1007.6*	ribosomal protein, small subunit	2.1	5.22E-03
	RPL-13	*c32e8.2*	60S ribosomal protein L13	2.0	7.94E-03
	RPS-11	*f40f11.1*	ribosomal protein, small subunit	1.7	7.59E-03
	RPS-1	*f56f3.5*	40S ribosomal protein S3a	1.6	8.48E-03
	RPS-28	*y41d4b.5*	40S ribosomal protein S28	1.4	9.56E-03
	MRPL-12	*w09d10.3*	mitochondrial ribosomal protein, large	2.5	5.09E-03
	RUVB-1	*c27h6.2*	RuvB-like 1	infected only	
	EIF-3.J	*y40b1b.5*	eukaryotic initiation factor	1.7	4.35E-03
	K07C5.4	*k07c5.4*	ortholog of human NOP56 ribonucleoprotein	1.5	9.73E-03
	SRP-6	*c03g6.19*	serpin (serine protease inhibitor)	infected only	
proteolysis/hydrolysis	KLO-1	*c50f7.10*	KLOtho (mammalian aging-associated protein) homolog	2.6	1.74E-03
	F13H6.3	*f13h6.3*	ortholog of human carboxylesterase 2	2.3	4.69E-03
	TAX-6	*c02f4.2*	Serine/threonine-protein phosphatase	2.0	8.34E-03
	Y25C1A.13	*y25c1a.13*	ortholog of human enoyl CoA hydratase 1	2.0	9.29E-03
	PAM-1	*f49e8.3*	puromycin-sensitive aminopeptidase	1.8	2.09E-03
	T22C1.6	*t22c1.6*	ortholog of human taxilin α, β, and γ	infected only	
ER/UPR	Y41C4A.11	*y41c4a.11*	involved in ER UPR	4.3	1.84E-03
	C14B9.2	*c14b9.2*	probable protein disulfide-isomerase A4	2.3	1.52E-03
	HSP-4	*f43e2.8*	heat shock protein	2.2	2.00E-03
	PDI-1	*c14b1.1*	protein disulfide isomerase	1.6	8.26E-03
	SNA-1	*w02f12.6*	snRNP-binding protein	infected only	
Transcription	HRP-1	*f42a6.7*	putative hnRNP	5.5	6.47E-05
	T13F2.2	*t13f2.2*	predicted to have transcription coactivator activity	3.3	1.74E-03
	RPB-2	*c26e6.4*	RNA polymerase II (B) subunit	3.1	3.22E-05
	RPB-9	*y97e10ar.5*	RNA polymerase II (B) subunit	2.5	6.86E-03
	CEY-4	*y39a1c.3*	*C. elegans* Y-box	2.0	1.05E-04
	CEY-2	*f46f11.2*	*C. elegans* Y-box	1.7	3.55E-03
	CPG-2	*b0280.5*	chondroitin proteoglycan	infected only	
Development/reproduction	C29E4.12	*c29e4.12*	ortholog of human C7orf55	infected only	
	Y18D10A.9	*y18d10a.9*	ortholog of human CIAO1	infected only	
	T23D8.3	*t23d8.3*	ortholog of human and yeast LTV1	infected only	
	K07H8.10	*k07h8.10*	predicted to have necleic acid and nucleotide binding activity	8.2	1.56E-05
	F55B11.2	*f55b11.2*	development	4.4	9.81E-03
	DNC-2	*c28h8.12*	dynactin complex component	3.2	3.30E-03
	F32A11.3	*f32a11.3*	involved in reproduction	3.2	9.82E-03
	Y71F9AL.9	*y71f9al.9*	ortholog of human SPATS2L and SPATS2	2.1	4.21E-04
	DPY-30	*zk863.6*	dosage compensation protein	2.0	4.34E-03
	PRX-19	*f54f2.8*	Peroxisome assembly factor	1.9	9.17E-03
	MRPS-28	*y43f8c.8*	Mitochondrial ribosomal Protein	1.8	1.63E-03
	C44E4.4	*c44e4.4*	ortholog of human Sjogren syndrome atigen B	1.6	7.28E-03
	F20D6.11	*f20d6.11*	putative FAD-binding oxidoreductase	infected only	
Cell redox homeostasis	F29C4.2	*f29c4.2*	ortholog of human cytochrome c oxidase subunit Vic	infected only	
	Y47G6A.21	*y47g6a.21*	predicted to have oxidoreductase activity	2.8	1.91E-03
	F45H10.3	*f45h10.3*	predicted to have NADH dehydrogenase (ubiquinone) activity	2.6	7.79E-03
	PRDX-6	*y38c1aa.11*	preoxiredoxin 6	1.7	6.03E-03
	KIN-10	*t01g9.6*	protein kinase	infected only	
Protein Kinase	CAT-4	*f32g8.6*	GTP cyclohydrolase 1	infected only	
	MMCM-1	*zk1058.1*	methylmalonyl-CoA mutase homolog	5.4	4.87E-03
compound metabolic process	C36A4.4	*c36a4.4*	probable UDP-N-acetylglucosamine pyrophosphorylase	2.7	4.17E-03
	C39D10.7	*c39d10.7*	involved in chitin metabolic process	2.0	6.91E-03
	ZK1307.8	*zk1307.8*	ortholog of human protein kinase C substrate 80K-H	1.8	8.37E-03
	CTS-1	*t20g5.2*	citrate synthase	1.7	4.81E-03
	DPYD-1	*c25f6.3*	dihydropyrimidine dehydrogenase	1.7	5.14E-03

^#^Infected only: the protein was only detected in the infected animals, not in the uninfected animals.
