# Supplementary material for: Neuronal GPCR OCTR-1 regulates innate immunity by controlling protein synthesis in Caenorhabditis elegans
Source: Sci Rep. 2016 Nov 11;6:36832. doi: 10.1038/srep36832 (PMC5104976; doi:10.1038/srep36832)
Supplement: Supplementary Information [file srep36832-s1.pdf]

Supplementary Information

Neuronal GPCR OCTR-1 regulates innate immunity by controlling protein synthesis in *Caenorhabditis elegans*

Yiyong Liu[1], Durai Sellegounder[1], and Jingru Sun[1]*

[1]Department of Biomedical Sciences, Elson S. Floyd College of Medicine, Washington State University, Spokane, Washington, USA

*Corresponding. jingru.sun@wsu.edu

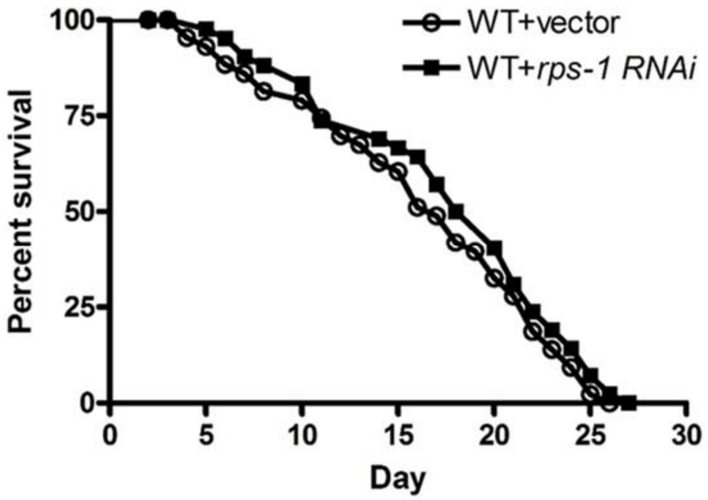

Figure S1: Wild-type animals treated with *rps-1* RNAi have a lifespan similar to that of wild-type animals treated with empty vector control RNAi. Wild-type animals grown on double-stranded RNA (dsRNA) for vector control or dsRNA for *rps-1* were scored for survival over time. WT+vector versus WT+*rps-1* RNAi, $p = 0.2879$. The survival graph represents assays of two independent experiments. $n = 45$ animals per strain.

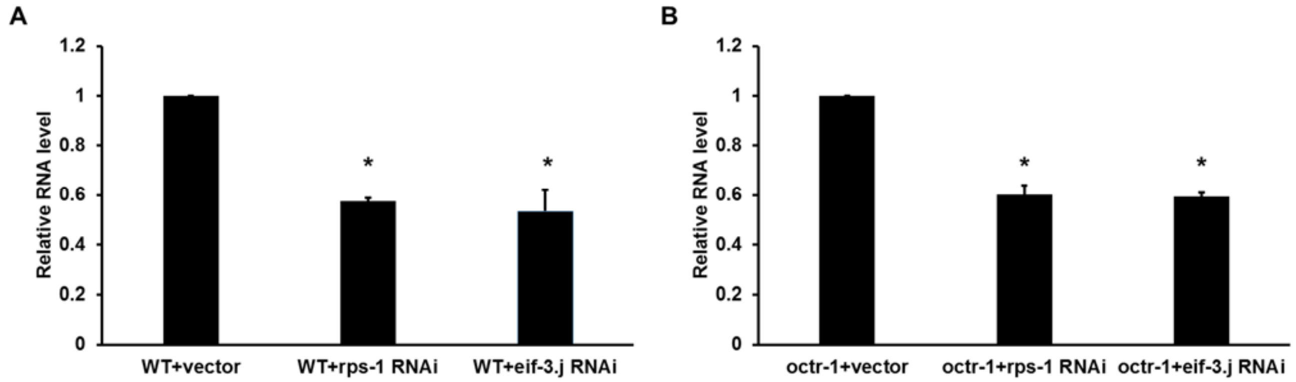

Figure S2: *rps-1* RNAi and *eif-3.j* RNAi in wild-type and *octr-1(ok371)* animals. (**A**) qRT-PCR analysis of expression of *rps-1* and *eif-3.j* in wild-type animals. Bars represent mean ± SEM. *n* = 3 independent experiments. * denotes a significant difference ($p < 0.001$) between the means of the WT+RNAi animals and the WT+vector animals. (**B**) qRT-PCR analysis of expression of *rps-1* and *eif-3.j* in *octr-1(ok371)* animals. Bars represent mean ± SEM. *n* = 3 independent experiments. * denotes a significant difference ($p < 0.001$) between the means of the octr-1+RNAi animals and the octr-1+vector animals.

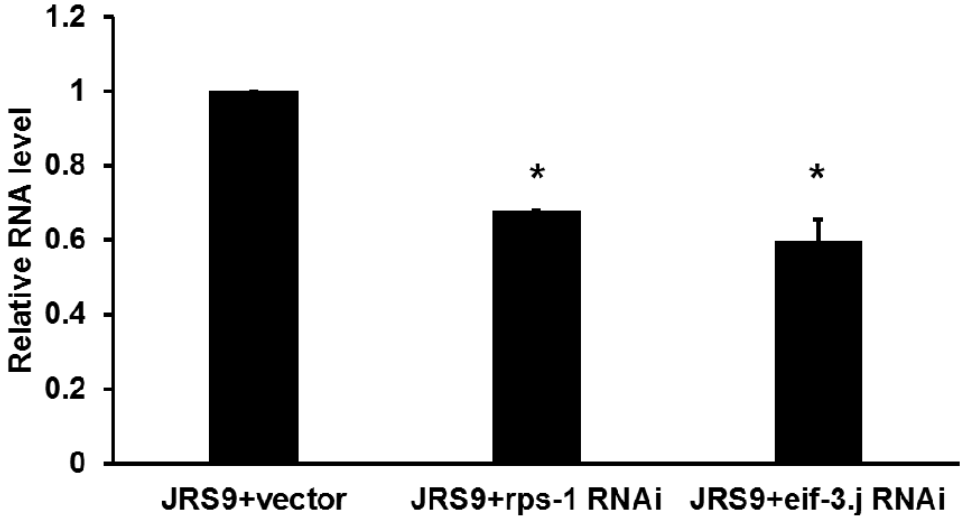

Figure S3: *rps-1* RNAi and *eif-3.j* RNAi in JRS9 (*octr-1(ok371);Phsp-4::GFP(zcls4)*) animals. qRT-PCR analysis of expression of *rps-1* and *eif-3.j* in JRS9 animals. Bars represent mean ± SEM. $n = 3$ independent experiments. * denotes a significant difference ($p < 0.001$) between the means of the JRS9+RNAi animals and the JRS9+vector animals.

Table S1. Proteins downregulated in wild-type N2 animals upon *P. aeruginosa* infection

**Protein**	**Gene ID**	**Fold Change#**	**p-value**
GRD-14	*t01b10.2*	only in the uninfected	
C05C8.7	*c05c8.7*	only in the uninfected	
GPX-5	*c11e4.1*	only in the uninfected	
HACD-1	*r09b5.6*	only in the uninfected	
Y54G11A.7	*y54g11a.7*	only in the uninfected	
Y43F8C.13	*y43f8c.13*	only in the uninfected	
Y49E10.18	*y49e10.18*	only in the uninfected	
BAF-1	*b0464.7*	only in the uninfected	
CLEC-5	*c35d10.14*	only in the uninfected	
SEC-24.2	*zc518.2*	only in the uninfected	
ZK1320.2	*zk1320.2*	only in the uninfected	
NDX-2	*w02g9.1*	only in the uninfected	
CYTB-5.2	*w02d3.1*	only in the uninfected	
THN-2	*f28d1.5*	only in the uninfected	
F28B4.3	*f28b4.3*	5.8	0.009336
C49C3.4	*C49c3.4*	3.2	0.00538

only in the uninfected: the protein was only detected in the uninfected wild-type N2 animals, not in the infected wild-type N2 animals.

Table S2. Proteins upregulated in *octr-1(ok371)* animals upon *P. aeruginosa* infection

Protein	Gene ID	Fold Change#	p-value
METR-1	*r03d7.1*	only in the infected	
DOD-24	*c32h11.12*	only in the infected	
F20D6.11	*f20d6.11*	only in the infected	
M60.2	*m60.2*	only in the infected	
F55G11.2	*f55g11.2*	only in the infected	
CPG-2	*b0280.5*	only in the infected	
SKPO-1	*f49e12.1*	only in the infected	
RUVB-1	*c27h6.2*	only in the infected	
F55G11.4	*f55g11.4*	only in the infected	
D1054.11	*d1054.11*	only in the infected	
C32H11.4	*c32h11.4*	only in the infected	
C29E4.12	*c29e4.12*	only in the infected	
C14C6.5	*c14c6.5*	only in the infected	
C33A12.1	*c33a12.1*	only in the infected	
Y18D10A.9	*y18d10a.9*	only in the infected	
T23D8.3	*t23d8.3*	only in the infected	
DOD-17	*k10d11.1*	only in the infected	
LYS-2	*y22f5a.5*	only in the infected	
C01G10.9	*c01g10.9*	only in the infected	
C29G2.6	*c29g2.6*	only in the infected	
HMG-11	*t05a7.4*	only in the infected	
C17H12.8	*c17h12.8*	only in the infected	
CAT-4	*f32g8.6*	only in the infected	
CLEC-67	*f56d6.2*	only in the infected	
CLEC-66	*f35c5.9*	only in the infected	
SRP-6	*c03g6.19*	only in the infected	
KIN-10	*t01g9.6*	only in the infected	
GST-38	*f35e8.8*	only in the infected	
SNA-1	*w02f12.6*	only in the infected	
T22C1.6	*t22c1.6*	only in the infected	
F29C4.2	*f29c4.2*	only in the infected	
CD4.3	*cd4.3*	only in the infected	
F35E12.6	*f35e12.6*	only in the infected	
IRG-3	*f53e10.4*	only in the infected	
GST-5	*r03d7.6*	9.5	0.006983
K07H8.10	*k07h8.10*	8.2	1.56E-05
PUD-1.1	*f15e11.13*	7.4	0.004199
ASP-14	*k10c2.3*	7.2	0.005772
HRP-1	*f42a6.7*	5.5	6.47E-05
MMCM-1	*zk1058.1*	5.4	0.004868

F55B11.2	*f55b11.2*	4.4	0.009808
Y41C4A.11	*y41c4a.11*	4.3	0.001836
PUD-2.1	*f15e11.1*	4.2	0.000795
CTH-2	*zk1127.10*	4.1	0.006442
GST-7	*f11g11.2*	3.9	0.009306
GLB-1	*zk637.13*	3.9	0.000373
CLEC-63	*f35c5.6*	3.5	0.006046
F59B1.2	*f59b1.2*	3.3	0.001724
T13F2.2	*t13f2.2*	3.3	0.001735
DNC-2	*c28h8.12*	3.2	0.0033
SDHA-2	*c34b2.7*	3.2	0.008798
F32A11.3	*f32a11.3*	3.2	0.009824
RPB-2	*c26e6.4*	3.1	3.22E-05
F28H7.3	*f28h7.3*	3.0	0.001857
Y47G6A.21	*y47g6a.21*	2.8	0.001912
GST-4	*k08f4.7*	2.8	0.009806
C36A4.4	*c36a4.4*	2.7	0.004171
GCS-1	*f37b12.2*	2.7	0.008723
KLO-1	*c50f7.10*	2.6	0.001736
F45H10.3	*f45h10.3*	2.6	0.007795
RPB-9	*y97e10ar.5*	2.5	0.00686
C42D4.1	*c42d4.1*	2.5	0.001481
Y45F10C.4	*y45f10c.4*	2.5	0.007357
F58H1.3	*f58h1.3*	2.5	8.81E-05
MRPL-12	*w09d10.3*	2.5	0.005091
SKR-3	*f44g3.6*	2.3	0.006315
F13H6.3	*f13h6.3*	2.3	0.00469
C14B9.2	*c14b9.2*	2.3	0.001525
CYSL-2	*k10h10.2*	2.3	0.000171
HSP-4	*f43e2.8*	2.2	0.001997
ATP-4	*t05h4.12*	2.2	0.005098
MBF-1	*h21p03.1*	2.2	0.007713
MTSS-1	*par2.1*	2.1	0.009034
RPL-18	*y45f10d.12*	2.1	0.005226
RPS-10	*d1007.6*	2.1	0.005219
C45B2.1	*c45b2.1*	2.1	0.001643
C27H6.8	*c27h6.8*	2.1	0.005811
H28O16.1	*h28o16.1*	2.1	0.001716
Y71F9AL.9	*y71f9al.9*	2.1	0.000421
CEY-4	*y39a1c.3*	2.0	0.000105
C39D10.7	*c39d10.7*	2.0	0.006913
DDP-1	*y39a3cr.4*	2.0	0.000334
TAX-6	*c02f4.2*	2.0	0.008339

DPY-30	*zk863.6*	2.0	0.004345
RPL-13	*c32e8.2*	2.0	0.007937
Y25C1A.13	*y25c1a.13*	2.0	0.009293
ATP-2	*c34e10.6*	2.0	0.001436
ZK418.9	*zk418.9*	1.9	0.002168
C18H9.3	*c18h9.3*	1.9	0.00231
Y39B6A.33	*y39b6a.33*	1.9	0.003169
PRX-19	*f54f2.8*	1.9	0.009167
MRPS-28	*y43f8c.8*	1.8	0.001627
ACDH-7	*t25g12.5*	1.8	0.003477
Y73E7A.1	*y73e7a.1*	1.8	0.004306
DJR-1.1	*b0432.2*	1.8	0.002247
SAMS-4	*c06e7.3*	1.8	0.009821
ZK1307.8	*zk1307.8*	1.8	0.008369
PAM-1	*f49e8.3*	1.8	0.002091
F42A10.5	*f42a10.5*	1.8	0.000797
MAI-2	*b0546.1*	1.8	0.000393
TIN-13	*dy3.1*	1.8	0.004899
CTS-1	*t20g5.2*	1.7	0.004813
RPS-11	*f40f11.1*	1.7	0.007592
DPYD-1	*c25f6.3*	1.7	0.005138
PRDX-6	*y38c1aa.11*	1.7	0.006034
VIG-1	*f56d12.5*	1.7	0.000787
EIF-3.J	*y40b1b.5*	1.7	0.004354
CEY-2	*f46f11.2*	1.7	0.003555
C44E4.4	*c44e4.4*	1.6	0.00728
PDI-1	*c14b1.1*	1.6	0.008259
RPS-1	*f56f3.5*	1.6	0.008484
K07C5.4	*k07c5.4*	1.5	0.009731
RPS-28	*y41d4b.5*	1.5	0.009558

only in the uninfected: the protein was only detected in the infected *octr-1(ok371)* animals, not in the uninfected *octr-1(ok371)* animals.

Table S3. Effects of knockdown of protein synthesis factors by RNAi on *C. elegans* development

RNAi Target*	Effect on wild-type worms	Effect on *octr-1(ok371)* worms
F56F3.5 *(rps-1)*	After L2 larval worms fed RNAi bacterial culture for 48 hrs, the worms were normal in egg laying and development compared to worms treated with vector control RNAi.	After L2 larval worms fed RNAi bacterial culture for 48 hrs, the worms were normal in egg laying and development compared to worms treated with vector control RNAi.
F40F11.1 *(rps-11)*	After L2 larval worms fed RNAi bacterial culture for 48 hrs, the worms displayed defective egg laying. 20 Gravid worms synchronized for 1 hr at 25°C only laid 20 eggs per worm in average while worms with empty vector control RNAi laid approximately 110 eggs per worm. The eggs from worms with *rps-11* RNAi were hatched very slowly, and development was arrested at L1 stage. Worms displayed immature oocyte.	After L2 larval worms fed RNAi bacterial culture for 48 hrs, the worms displayed defective egg laying. 20 Gravid worms synchronized for 1 hr at 25°C only laid 24 eggs per worm in average while worms with empty vector control RNAi laid approximately 110 eggs per worm. The eggs from worms with *rps-11* RNAi were hatched very slowly, and development was arrested at L1 stage. Worms displayed immature oocyte.
W09D10.3 *(mrpl-12)*	After L2 larval worms fed RNAi bacterial culture for 48 hrs, the worms displayed normal in egg laying phenotype. These eggs hatched normally but their development was arrested at L1 stage.	After L2 larval worms fed RNAi bacterial culture for 48 hrs, the worms displayed normal in egg laying phenotype. These eggs hatched normally but their development was arrested at L1 stage.
C32E8.2 *(rpl-13)*	After L2 larval worms fed RNAi bacterial culture for 48 hrs, the worms displayed defective egg laying phenotype. 20 Gravid worms synchronized for 1 hr at 25°C only laid 12 eggs per worm in average. None of the laid eggs hatched.	After L2 larval worms fed RNAi bacterial culture for 48 hrs, the worms displayed defective egg laying phenotype. 20 Gravid worms synchronized for 1 hr at 25°C only laid 14 eggs per worm in average. None of the laid eggs hatched.
Y45F10D.12 *(rpl-18)*	After L2 larval worms fed RNAi bacterial culture for 48 hrs, the worms displayed normal in egg laying phenotype. However, few eggs hatched and development was arrested at L1-L2 stages.<br>Worms displayed dumpy phenotype compared with worms treated with vector RNAi at same development stage.	After L2 larval worms fed RNAi bacterial culture for 48 hrs, the worms displayed normal in egg laying phenotype. However, few eggs hatched and development was arrested at L1-L2 stages.<br>Worms displayed dumpy phenotype compared with worms treated with vector RNAi at same development stage.
D1007.6 *(rps-10)*	After L2 larval worms fed RNAi bacterial culture for 48 hrs, the worms displayed normal in egg laying and development compared with worms treated with vector control RNAi.	After L2 larval worms fed RNAi bacterial culture for 48 hrs, the worms displayed normal in egg laying and development compared with worms treated with vector control RNAi.

K07C5.4	After L2 larval worms fed RNAi bacterial culture for 48 hrs, the worms displayed normal in egg laying phenotype. Few eggs were hatched, and development was arrested at L1-L2 stages.	After L2 larval worms fed RNAi bacterial culture for 48 hrs, the worms displayed normal in egg laying phenotype. Few eggs were hatched, and development was arrested at L1-L2 stages.
C27H6.2 (*ruvb-1*)	After L2 larval worms fed RNAi bacterial culture for 48 hrs, the worms displayed normal in egg laying phenotype. These eggs hatched normally but their development was arrested at L1 stage.	After L2 larval worms fed RNAi bacterial culture for 48 hrs, the worms displayed normal in egg laying phenotype. These eggs hatched normally but their development was arrested at L1 stage.
Y40B1B.5 *(eif-3.j)*	After L2 larval worms fed RNAi bacterial culture for 48 hrs, the worms displayed normal in egg laying and development compared with worms treated with vector control RNAi.	After L2 larval worms fed RNAi bacterial culture for 48 hrs, the worms displayed normal in egg laying and development compared with worms treated with vector control RNAi.

* Y41D4B.5 (*rps-28*) RNAi clone is not available in either Ahringer *C. elegans* RNAi library or Geneservice *C. elegans* RNAi library.
